# HBV infection and pregnancy: Increased risks of gestational diabetes and preterm birth

**DOI:** 10.12669/pjms.41.4.9578

**Published:** 2025-04

**Authors:** Xiumei Cai, Suiping Huang, Zuliang Ma

**Affiliations:** 1 Xiumei Cai, Department of Medical Quality Control, Nanhai Affiliated Maternity and Children’s Hospital of, Guangzhou University of Chinese Medicine, Foshan, China; 2 Suiping Huang, Department of Disease Prevention and Health Care, Shunde Hospital, Southern Medical University, The First People’s Hospital of Shunde, Foshan, China; 3 Zuliang Ma, Department of Disease Prevention and Health Care, Shunde Hospital, Southern Medical University, The First People’s Hospital of Shunde, Foshan, China

**Keywords:** HBV infection, Pregnancy outcomes

## Abstract

**Objective:**

To investigate the effects of Hepatitis B virus (HBV) infection status on the outcomes of pregnancy.

**Methods:**

A retrospective study was conducted at Shunde Hospital of Southern Medical University (The First People’s Hospital of Shunde) from 1^st^ January 2020 to 31^st^ December2022.The study included 13,980 pregnant women, with 1,059 in the HBsAg-positive group (study group) and 12,921 in the HBsAg-negative group (control group). Pregnancy outcomes were compared between the control and study groups.

**Results:**

Pregnant women who tested positive for HBsAg had higher rates of gestational diabetes mellitus (P=0.019; OR, 1.20; 95% CI, 1.03-1.40), intrahepatic cholestasis of pregnancy (P=0.019; OR, 1.85; 95% CI, 1.11-3.09) and preterm birth before 34 weeks (P=0.013; OR, 1.96; 95% CI, 1.15-3.34). The relationship between HBV infection status with gestational diabetes mellitus, intrahepatic cholestasis of pregnancy and preterm birth before 34 weeks was significant.

**Conclusions:**

Pregnant women who tested positive for HBsAg faced a higher likelihood of developing gestational diabetes mellitus, intrahepatic cholestasis of pregnancy, and giving birth prematurely before 34 weeks. Further clarification is required regarding the influence of HBV infection on the outcomes of pregnancy.

## INTRODUCTION

The infection of the Hepatitis B virus (HBV) poses a major concern for public health, resulting in a substantial healthcare burden worldwide. Approximately 296 million individuals worldwide are currently affected by chronic hepatitis B virus (CHB) infection.^1^ Most of them reside in the Asia Pacific region, with China having the highest number of HBV cases, affecting 74.6 million individuals.^2^ Additionally, the prevalence of HBV among pregnant Chinese women was remarkably high, reaching 6.64%.^3^

A systematic review indicates that HBV infection can lead to an increase in pro-inflammatory cytokines in pregnant women, thereby affecting insulin regulation and promoting the secretion of oxytocin by uterine smooth muscle cells, which in turn increases the risk of gestational diabetes mellitus (GDM) and preterm birth; HBV infection can also enhance the compensatory synthesis of bile acids through the HBV viral envelope protein Pre-S1.^4^

Nevertheless, the uncertainty persists regarding the impact of maternal HBV infection on the likelihood of unfavorable pregnancy results. For instance, study indicated that expectant mothers with HBV infection had an increased likelihood of developing gestational diabetes mellitus (GDM).^5^Nevertheless, the findings did not align with the outcomes of other studies.^6^, ^7^ Furthermore, research indicated a decreased likelihood of preeclampsia in expectant mothers with HBV^8^, but several other studies did not find such a relationship.^6^, ^7^

Moreover, the majority of research utilized data from nations with a low occurrence of pregnant women infected with HBV. Hence, the results might not be applicable to more vulnerable nations such as China. Therefore, in order to evaluate the situation in our country, we carried out this retrospective cohort investigation to examine the correlation between pregnant women with HBV and unfavorable pregnancy results among the Chinese populace.

## METHODS

**Fig.1:**
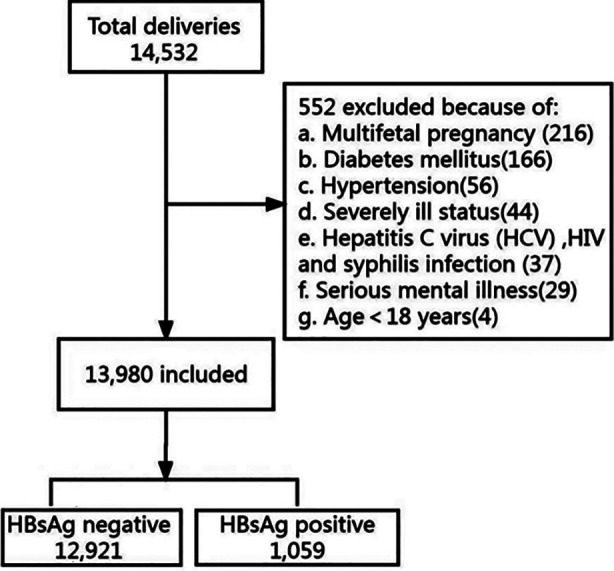
Flow chart for study population.

We analyzed 14,532 women who delivered at the Shunde Hospital of Southern Medical University, China, during the time frame of January 1, 2020 to December 31, 2022. Amongst these, 552 subjects were excluded due to age<18 years; multiple pregnancy; severely ill status; serious mental illness; concurrent medical complications and other infection. Finally, 13,980 pregnant women were enrolled in this study (Fig.1). All pregnant women attending the antenatal clinic undergo routine blood tests to screen for HBsAg using enzyme-linked immunosorbent assay (ELISA). The HBsAg status of each pregnant woman is documented in their medical records. HBV infection was defined as HBsAg seropositivity.^9^ The HBV-positive pregnant women were assigned to the study group, whereas the control group included the remaining pregnancies.

### Inclusion criteria:

- Age≥18 years.
- Singleton pregnancy.
- Demographic information, medical records, and prenatal test results meet the study’s needs.

### Exclusion criteria:

- Pre-existing hypertension or diabetes prior to pregnancy.
- With other viral hepatitis or infections such as HIV, syphilis.
- Severe liver diseases such as cirrhosis, liver cancer.
- Concurrent malignant tumors, immunodeficiency diseases.
- Severely ill status.
- Serious mental illness.

### Ethical Approval:

This study was approved by the Medical Ethics Committee of Southern Medical University (The First People’s Hospital of Shunde) (No: KYLS20220794), July 22, 2022.

### Outcome measures:

The following data were obtained from the hospital’s medical record management system:

- Information related to pregnancy and delivery, such as age, gestational weeks, gravidity, parity, mode of delivery, birth weight, HBsAg status, ethnicity, profession, marital status, in vitro fertilization(IVF) and gestational anemia;
- Details regarding pregnancy outcomes and maternal complications, including gestational hypertension, preeclampsia, postpartum hemorrhage, GDM, placenta previa, Apgar score, obesity, intrahepatic cholestasis of pregnancy (ICP), placental abruption and premature rupture of membranes (PROM).

### Statistical analyses:

It was conducted using SPSS 26.0 (SPSS Inc, Chicago, IL, USA). Mean ± SD was used to express continuous variables and the t-test was used to analyze variations between the study group and control group. Categorical variables were expressed as number or percent and tested by the χ^2^ test. Additionally, relative risks (OR) were calculated with 95% CI. Multivariate logistic regression analysis was performed to examine the relationship between the HBV infection and pregnancy outcomes. A significance level for p-value was 0.05 or lower was considered as statistical significance.

## RESULTS

### Baseline characteristics:

The subjects’ ages ranged from 18 to 50 years, with 41 participants (0.29%) under 20 years old, 847 (6.06%) aged 20-25, 4605 (32.94%) aged 25-30, 5448 (38.97%) aged 30-35, and 3039 (21.74%) aged 35 and above. In Table-I, the epidemiological features of 13,980 participants in the study. Out of the total number of women, 1,059 were found to be HBsAg-positive, while the control group consisted of 12,921 HBsAg-negative women. The average age of the study group was higher compared to the control group (34.2±4.8 vs. 31.8±4.5, *P*<0.001). The two groups showed notable variations in gravidity, parity and mode of delivery. On the other hand, there were no notable disparities in obesity rates between the two groups.

**Table-I:** Epidemiological characteristics with respect to HBsAg status.

Parameters	HBsAg-positive n = 1,059 (%)	HBsAg-negative n = 12,921 (%)	χ^2^/t	p-Values
Age (years)	34.2±4.8	31.8±4.5	16.27	<0.001^a,***^
Gravidity	-	-	-	-
1	244/1,059(23.04)	4,305/12,921(33.32)	47.10	<0.001^b,***^ -
≥2	815/1,059(76.96)	8,616/12,921(66.68)	-	
Parity	-	-	-	-
1	342/1,059(32.29)	5,778/12,921(44.72)	61.38	<0.001^b,***^ -
≥2	717/1,059(67.71)	7,143/12,921(55.28)	-	
Obesity	-	-	-	-
Yes	34/1,059(3.21)	386/12,921(2.99)	0.17	0.683^b^ -
No	1,025/1,059(96.79)	12,535/12,921(97.01)	-	
Delivery mode	-	-	-	-
Vaginal	660/1,059(62.32)	8,552/12,921(66.19)	6.50	0.011^b,**^ -
Caesarean section	399/1,059(37.68)	4,369/12,921(33.81)	-	
Ethnicity	-	-	-	-
Han	1,044/1,059(98.58)	12,720/12,921(98.44)	0.13	0.724^b^
Others	15/1,059(1.42)	201/12,921(1.56)	-	-
Profession	-	-	-	-
Employee	602/1,059(56.85)	7,665/12,921(59.32)	2.48	0.115^b^
Housewife/unemployed	457/1,059(43.15)	5,25612,921(40.68)	-	-
Marital status	-	-	-	-
Married	1,043/1,059(98.49)	12,617/12,921(97.65)	3.10	0.078^b^
Unmarried/Divorced	16/1,059(1.51)	304/12,921(2.35)	-	-
In vitro fertilization	-	-	-	-
Yes	37/1,059(3.49)	421/12,921(3.26)	0.17	0.679^b^
No	1,022/1,059(96.51)	12,500/12,921(96.74)	-	-
Gestational anemia	-	-	-	-
Yes	334/1,059(31.54)	3,858/12,921(29.86)	1.32	0.251^b^
No	725/1,059(68.46)	9,063/12,921(70.14)	-	-

*Note*:1. Results expressed in number (%) and compared with χ^2^ test, or in mean ± standard deviation (SD) and compared with t test. 2. ‘a’ indicates t-test data, and ‘b’ indicates chi square data.3. ‘*’ indicates 0.01 ≤*p*< 0.05, ‘**’ indicates 0.001 ≤*p* < 0.01, and ‘***’ indicates *p*<0.001. 4. ‘-’indicates no data.

### Pregnancy outcomes:

No notable disparities were noted in terms of pregnancy complications between the two groups, including gestational hypertension (*P*=0.296; OR, 0.71; 95% CI, 0.38-1.35), PROM (*P*=0.649; OR, 0.97; 95% CI, 0.83-1.13), preeclampsia (*P*=0.969; OR, 0.99; 95% CI, 0.67-1.48) and placenta previa (*P*=0.251; OR, 1.34; 95% CI, 0.81-2.23). The incidence of postpartum hemorrhage was higher in the study group, but the difference was not statistically (4.06% vs 3.10%; *P*=0.089; OR, 1.32; 95% CI, 0.96-1.82). No significant distinction was observed in neonatal outcomes, including low birth weight (*P*=0.217; OR, 1.18; 95% CI, 0.91-1.41), macrosomia (*P*=0.809; OR, 0.95; 95% CI, 0.62-1.45), Apgar score<8 (1 min) (*P*=0.228; OR, 0.68; 95% CI, 0.36-1.28), between the two groups.

Within the study group, there was a notably greater occurrence of GDM (*P*=0.019; OR, 1.20; 95% CI, 1.03-1.40). In the meantime, the occurrence of ICP was twice as high in the study group (*P*=0.019; OR, 1.85; 95% CI, 1.11-3.09). Specifically, there was no notable disparity in the occurrence of birth before 37 weeks (*P*=0.052; OR, 1.30; 95% CI, 1.00-1.69). Nevertheless, further analysis showed the incidence of birth before 34 weeks was two-fold higher in the study group (*P*=0.013; OR, 1.96; 95% CI, 1.15-3.34).

Multivariable analysis showed that the HBV infection was remained a significant risk factor for GDM (*P*=0.023; adjusted OR, 1.19; 95% CI, 1.03-1.39), ICP (*P*=0.015; adjusted OR, 1.91; 95% CI, 1.13-3.22) and birth before 34 weeks (*P*=0.015; adjusted OR, 1.95; 95% CI, 1.14-3.33) after adjusting for confounding factors including age, gravidity and parity. Relevant data are presented in Table-II.

**Table-II:** Pregnancy outcomes with respect to HBsAg status.

Parameters	HBsAg-positive n = 1,059 (%)	HBsAg-negative n = 12,921 (%)	Crude OR(95% CI)	p-Values	Adjusted OR (95% CI)	p-Values
Gestational weeks	-	-	-	-	-	-
<34 weeks	16(1.51)	101(0.78)	1.96(1.15-3.34)	0.013	1.95(1.14-3.33)	0.015*
<37 weeks	66(6.23)	630(4.88)	1.30(1.00-1.69)	0.052	1.19(0.92-1.56)	0.191
Birth weight	-	-	-	-	-	-
Low birth weight	63(5.95)	656(5.08)	1.18(0.91-1.54)	0.217	1.20(0.91-1.57)	0.196
Macrosomia	24(2.27)	308(2.38)	0.95(0.62-1.45)	0.809	0.88(0.57-1.35)	0.555
Gestational hypertension	10(0.94)	171(1.32)	0.71(0.38-1.35)	0.296	0.60(0.32-1.15)	0.124
GDM	230(21.72)	2427(18.78)	1.20(1.03-1.40)	0.019	1.19(1.03-1.39)	0.023*
PROM	216(20.40)	2712(20.99)	0.97(0.83-1.13)	0.649	1.00(0.86-1.18)	0.975
Preeclampsia	27(2.55)	332(2.57)	0.99(0.67-1.48)	0.969	0.94(0.63-1.41)	0.763
ICP	17(1.61)	113(0.87)	1.85(1.11-3.09)	0.019	1.91(1.13-3.22)	0.015*
Postpartum hemorrhage	43(4.06)	401(3.10)	1.32(0.96-1.82)	0.089	1.26(0.91-1.76)	0.161
Placenta previa	17(1.61)	155(1.20)	1.34(0.81-2.23)	0.251	1.08(0.64-1.81)	0.773
Apgar score < 8 (1 min)	10(0.94)	180(1.39)	0.68(0.36-1.28)	0.228	0.62(0.32-1.18)	0.144

*Note:* 1. Results expressed in number (%) and compared with logistic regression analysis. 2.GDM, gestational diabetes mellitus; PROM, premature rupture of membranes; ICP, intrahepatic cholestasis of pregnancy. 3.’*’ indicates 0.01 ≤*p*< 0.05, ‘-’indicates no data.

## DISCUSSION

Our study uncovered a connection between HBV infection and a heightened likelihood of ICP, GDM and gestational weeks < 34 weeks. The connection between HBV infection and GDM is still a topic of debate. Several studies reported that the incidences of GDM were similar among HBsAg-negative women and HBsAg-positive women.^6^,^10^ Our result aligns with several studies that have demonstrated a higher risk of GDM in women who are HBsAg-positive.^11^, ^12^ Our research revealed that HBsAg-positive women had a slightly elevated risk of developing GDM in comparison to HBsAg-negative women (*P*=0.019; OR, 1.20; 95% CI, 1.03-1.40). The conflicting results may be involved multiple factors, including dietary patterns, the prevalence of HBV and diverse ethnicities.^13^-^15^ Several studies suggest that the increased risk of GDM may be attributed to the high occurrence of HBV infection^16^, taking into account the association between HBV infection and Type-2 diabetes mellitus.^17^ It is worth noting that Asians (including Chinese) have been found to have a significant occurrence of HBV infection^18^, as well as a high frequency of GDM based on studies conducted in multiethnic populations.^19^Another study indicate that GDM is more likely to occur in women with maternal HBV infection^20^, possibly because of the superimposed effect of pregnancy in the HBsAg-positive women. In addition, being of Asian race could potentially contribute as a separate risk factor for GDM.^21^ Therefore, further research is necessary to comprehend the correlation between GDM and HBV infection among pregnant women.

Various mechanisms may be responsible for the occurrence of GDM in females with HBV infection. Firstly, the increased risk of GDM in pregnant women with HBV infection may be due to the rise in pro-inflammatory cytokines, specifically tumor necrosis factor *alpha* (TNF-α), which was observed to spontaneously elevate in individuals with HBV infection^22^. TNF-α contributes to the risk of GDM by inhibiting insulin signaling and increasing glucose uptake regulated by insulin, thereby inducing insulin resistance during pregnancy.^23^, ^24^Secondly, high ferritin levels are thought to be associated with the development of GDM. The occurrence of elevated ferritin levels was higher in HBsAg-positive women, whereas elevated ferritin levels can impact the production and release of insulin, as well as enhance insulin resistance mediated by the liver.^20^ Thirdly, the liver, which has a crucial function in maintaining glucose homeostasis, can be adversely affected by HBV, resulting in glycometabolic dysfunction.^25^

Additionally, it was observed in this research that HBsAg-positive women had a higher likelihood of developing ICP in comparison to HBsAg-negative women (*P*=0.019; OR, 1.85; 95% CI, 1.11-3.09). Modifying the functions of various immune cells, such as natural killer cells and T cells, HBV can cause abnormalities in liver function and bile acid metabolism.^26^ In addition, HBV might enhance the likelihood of ICP through the utilization of Na^+^-taurocholic acid cotransporting polypeptide (NTCP), which acts as a functional receptor for HBV-infected hepatocytes and serves as the primary hepatic transporter for conjugated bile acids.^27^, ^28^HBV restricts the function of bile acid transporter by binding its envelope protein Pre-S1 to the NTCP, thereby promoting compensatory synthesis of bile acids.^29^, ^30^The effect of NTCP may change alone with the change of hormone levels during pregnancy.

HBsAg-positive women were also with an increased risk of delivery before 34 weeks(*P*=0.013; OR, 1.96; 95% CI, 1.15-3.34), aligning with findings from a previous investigation.^31^ Firstly, the increased risk of preterm labor in HBsAg-positive women is believed to be caused by the chronic inflammatory state^32^, as inflammation is an important risk factor for preterm labor.^33^ HBV infection leads to an increase in pro-inflammatory cytokines (interleukin-6(IL-6) and TNF-α) in pregnant women. IL-6 and TNF-α may increase the risk of preterm birth by inducing the production of prostaglandins in amniotic and decidual cells,^34^ activating matrix metalloproteinases,^35^ and enhancing the secretion of oxytocin by uterine smooth muscle cells.^36^ HBV infection can affect inflammation regulation by causing liver dysfunction, because the liver is a major organ that regulates inflammation through different mechanisms.^37^ Secondly, the accumulation of HBV-DNA in the placenta and trophoblastic cells can potentially trigger an inflammatory reaction, posing a risk for preterm labor.^7^

### Strengths:

We had a considerable sample size, which is expected to produce dependable findings. Secondly, the investigators were carefully selected based on specific criteria for inclusion and exclusion, aiming to minimize the potential bias.

### Limitations:

Firstly, this study did not include certain potential variables that could have influenced pregnancy outcomes. Despite regression models were used to control for potential confounders, confounding effects from unmeasured may persist. For example, smoking may be risk factor for adverse pregnancy outcomes. Due to the inherent limitations of retrospective studies, prospective research are needed in future research. Secondly, our analysis solely focused on the correlation between being HBsAg positive and pregnancy outcomes, without investigating the connection between other markers of HBV infection, like HBeAg, and the pregnancy outcomes. Our subsequent study will focus on examining how HBeAg impact the outcomes of pregnancy.

## CONCLUSION

In this retrospective investigation conducted in China, it has been observed that pregnant woman who test positive for HBsAg might encounter a heightened susceptibility to GDM, ICP and gestational weeks < 34 weeks. Considering the differences in HBV infection rate and ethnicity in different countries and regions, additional research is required to examine how viral characteristics and other clinical aspects of the disease affect the pregnancy outcomes.

### Authors’ Contributions:

**ZLM:** Funding acquisition, Conceptualization, Supervision, Writing-review and editing.

**XMC:** Data analysis, Writing-original draft, Writing-review and editing.

**SPH:** Data acquisition, Data analysis and Writing-original draft.

**ZLM:** Responsible and accountable for the accuracy and integrity of the study.

All authors have approved the final version to be published.

## References

[1] Global progress report on HIV, viral hepatitis, and sexually transmitted infections 2021 WHO.

[2] Guiying C, Wenzhan J, Jue L, Min L (2022). Countdown on hepatitis B elimination by 2030:the global burden of liver disease related to hepatitis B and association with socioeconomic status. Hepatol Int.

[3] Liu D, Liu Y, Ni J, Li H, Zeng L, Zhang C (2022). Hepatitis B Infection Among Pregnant Women in China:A Systematic Review and Meta-Analysis. Front Public Health.

[4] Cai WQ, Mao KY, Jiang PY, Zhou Y, Chen FL, Li D (2024). Correlation between hepatitis B virus infection and adverse pregnancy outcomes-a systematic review and meta-analysis. Zhonghua Gan Zang Bing Za Zhi.

[5] Yin W, Chen B, Yang Y, Li X, Li R, Xie J (2021). Association between maternal hepatitis B virus carrier and gestational diabetes mellitus:a retrospective cohort analysis. Virol J.

[6] Lobstein S, Faber R, Tillmann HL (2010). Prevalence of Hepatitis B among Pregnant Women and Its Impact on Pregnancy and Newborn Complications at a Tertiary Hospital in the Eastern Part of Germany. Digestion.

[7] Sirilert S, Traisrisilp K, Sirivatanapa P, Tongsong T (2014). Pregnancy outcomes among chronic carriers of hepatitis B virus. Int J Gynaecol Obstet.

[8] Lao TT, Sahota DS, Cheng YK, Law LW, Leung TY (2013). Maternal hepatitis B surface antigen status and incidence of pre-eclampsia. J Viral Hepat.

[9] Obstetrics Subgroup, Chinese Society of Obstetrics and Gynecology, Chinese Medical Association, Chinese Society of Perinatal Medicine, Chinese Medical Association (2020). 2020 Clinical guidelines on prevention of mother-to-child transmission of hepatitis B virus. Zhonghua Fu Chan Ke Za Zhi.

[10] Zhang Y, Chen J, Liao T, Chen S, Yan J, Lin X (2020). Maternal HBsAg carriers and pregnancy outcomes:a retrospective cohort analysis of 85,190 pregnancies. BMC Pregnancy Childbirth.

[11] Zhao Y, Chen YL, Song HQ, Huang PY, Li XJ (2020). Effects of maternal hepatitis B surface antigen positive status on the pregnancy outcomes:A retrospective study in Xiamen, China, 2011-2018. Plos One.

[12] Lao TT, Chan BC, Leung WC, Ho LF, Tse KY (2007). Maternal hepatitis B infection and gestational diabetes mellitus. J Hepatol.

[13] Atlaw D, Sahiledengle B, Assefa T, Negash W, Tahir A, Regasa T (2022). Incidence and risk factors of gestational diabetes mellitus in Goba town, Southeast Ethiopia:a prospective cohort study. BMJ Open.

[14] Nadeem S, Khatoon A, Rashid S, Ali F (2022). Dietary Intake patterns in women with GDM and Non-GDM:A comparative study. Pak J Med Sci.

[15] Zhang C, Jing L, Wang J (2023). Does depression increase the risk of gestational diabetes mellitus?A systematic review and meta-analysis. Pak J Med Sci.

[16] Terence TL, Ben CPC, Wing-Cheong L (2007). Maternal hepatitis B infection and gestational diabetes mellitus. J Hepatol.

[17] Knobler H, Schihmanter R, Zifroni A, Fenakel G, Schattner A (2000). Increased risk of type 2 diabetes in noncirrhotic patients with chronic hepatitis C virus infection. Mayo Clin Proc.

[18] Ma J, Bauman A (2010). Obstetric Profiles and Pregnancy Outcomes of Immigrant Women in New South Wales, 1990–1992. Australian New Zealand J Obstet Gynaecol.

[19] Willer KA, Winhofer Y, Kiss H, Falcone V, Berger A, Lechleitner M (2023). Gestational diabetes mellitus. Wien Klin Wochenschr.

[20] Lao TT, Tse KY, Chan LY, Tam KF, Ho LF (2003). HBsAg carrier status and the association between gestational diabetes with increased serum ferritin concentration in Chinese women. Diabetes Care.

[21] Chen L, Shi L, Zhang D, Chao SM (2019). Influence of Acculturation on Risk for Gestational Diabetes Among Asian Women. Prev Chronic Dis.

[22] Bekçibaşi M, Deveci Ö, Oğuz A, Bozkurt F, Dayan S, Çelen MK (2021). Serum TNF-α, IL-1β, and IL-6 levels in chronic HBV-infected patients. Int J Clin Pract.

[23] Tai TY, Lu JY, Chen CL, Lai MY, Chen PJ, Kao JH (2003). Interferon-alpha reduces insulin resistance and beta-cell secretion in responders among patients with chronic hepatitis B and C. J Endocrinol.

[24] Kampmann U, Knorr S, Fuglsang J, Ovesen P (2019). Determinants of Maternal Insulin Resistance during Pregnancy:An Updated Overview. J Diabetes Res.

[25] Raddatz D, Ramadori G (2007). Carbohydrate metabolism and the liver:actual aspects from physiology and disease. Z Gastroenterol.

[26] Dupuy FS, Durantel D, Lucifora J (2018). Liver macrophages:Friend or foe during hepatitis B infection?. Liver Int.

[27] Wang H, Shang X, Wan X, Xiang X, Mao Q, Deng G (2016). Increased hepatocellular carcinoma risk in chronic hepatitis B patients with persistently elevated serum total bile acid:a retrospective cohort study. Sci Rep.

[28] Shimura S, Watashi K, Fukano K, Peel M, Sluder A, Kawai F (2017). Cyclosporin derivatives inhibit hepatitis B virus entry without interfering with NTCP transporter activity. J Hepatol.

[29] Oehler N, Volz T, Bhadra OD, Kah J, Allweiss L, Giersch K (2014). Binding of hepatitis B virus to its cellular receptor alters the expression profile of genes of bile acid metabolism. Hepatology.

[30] König A, Döring B, Mohr C, Geipel A, Geyer J, Glebe D (2014). Kinetics of the bile acid transporter and hepatitis B virus receptor Na+/taurocholate cotransporting polypeptide (NTCP) in hepatocytes. J Hepatol.

[31] Tse KY, Lai FH, Lao T (2005). The impact of maternal HBsAg carrier status on pregnancy outcomes:a case-control study. J Hepatol.

[32] Zhang Y, Chen J, Liao T, Chen S, Yan J, Lin X (2020). Maternal HBsAg carriers and pregnancy outcomes:a retrospective cohort analysis of 85,190 pregnancies. BMC Pregnancy Childbirth.

[33] Menon R, Behnia F, Polettini J, Richardson LS (2020). Novel pathways of inflammation in human fetal membranes associated with preterm birth and preterm pre-labor rupture of the membranes. Semin Immunopathol.

[34] Wei YJ, Hs R, Lin YC, Won TW, Kan CD, Wang JN (2022). The Association of Patent Ductus Arteriosus with Inflammation:A Narrative Review of the Role of Inflammatory Biomarkers and Treatment Strategy in Premature Infants. Int J Mol Sci.

[35] Vadillo-Ortega F, Estrada-Gutiérrez G (2005). Role of matrix metalloproteinases in preterm labour. BJOG.

[36] Vilotić A, Nacka-Aleksić M, Pirković A, Bojić-Trbojević Ž, Dekanski D, JovanovićKrivokuća M (2022). IL-6 and IL-8:An Overview of Their Roles in Healthy and Pathological Pregnancies. Int J Mol Sci.

[37] Marra F, Tacke F (2014). Roles for chemokines in liver disease. Gastroenterology.

